# Totally extraperitoneal laparoscopic inguinal hernia repair post-radical prostatectomy

**DOI:** 10.1007/s00464-022-09281-z

**Published:** 2022-05-17

**Authors:** Imogen Watt, Adam Bartlett, John Dunn, Andrew Bowker

**Affiliations:** 1 Department of Surgery, University of Auckland, Auckland, New Zealand; 2 Laparoscopy Auckland, Gillies Ave, Epsom, Auckland, New Zealand; 3 Department of General Surgery, Auckland City Hospital, Park Road, Auckland, New Zealand

**Keywords:** Radical prostatectomy, Laparoscopic hernia repair, Totally extraperitoneal trans-abdominal preperitoneal, Laparoscopic inguinal hernia repair

## Abstract

**Background:**

Previous radical prostatectomy (RP) is considered a relative contraindication to the laparoscopic approach for inguinal hernia repair (LIHR). This study aimed to compare feasibility, safety and outcomes for patients undergoing totally extraperitoneal (TEP) LIHR who have previously undergone RP.

**Methods:**

This single surgeon, case–control study was performed using a prospective database of all patients undergoing TEP LIHR between 1995 and 2020. Patients who underwent previous RP were identified and compared to matched controls. Pre-operative, operative and post-operative data were analysed. The type of RP, open, laparoscopic or robotic, was identified and operative outcomes compared between the three groups.

**Results:**

6532 LIHR cases were identified. 165 had previously undergone RP and 6367 had undergone primary LIHR without prior RP. The groups were matched for age, demographics and co-morbidities. All operations were commenced laparoscopically, three converted to open in the LIHR + RP group and none in the LIHR group. Median operative time in patients with previous RP was longer, for unilateral (40 min vs. 21 min, *p* < 0.0001) and bilateral (71 vs. 30 min, *p* < 0.0001) LIHR. The majority of cases were performed as day stay procedures. There was no difference in immediate recovery parameters including time to discharge, complication rates, return to normal function, return to driving or post-operative analgesia. At 3 months of follow-up there was no difference in hernia recurrence for unilateral (2/128 vs 6/2234, *p* = 0.0658) or bilateral (0/24 vs 3/1490, *p* ≥ 0.999) LIHR, nor chronic pain as measured by patient awareness or restriction of activity. No differences in operative and post-operative outcomes were identified between the three types of RP, other than difference in operative time (*p* = 0.0336).

**Conclusions:**

Previous RP should not be an absolute contraindication for TEP LIHR. Although previous RP adds complexity, in experienced hands TEP LIHR can be done safely, with outcomes equivalent to patients who have not previously undergone RP.

Prostate cancer is one of the most commonly diagnosed cancers in men and for localised cancer, RP is the treatment of choice [1]. RP can either be performed by an open, laparoscopic or robot-assisted approach, and involves dissection through the preperitoneal space to resect the prostate, seminal vesicles and, in some cases, the adjacent lymph nodes [1, 2]. RP increases the risk of developing IH, with 7–21% of patients post RP developing IH [2–5]. RP disrupts the preperitoneal plane and consequently increases the complexity of a laparoscopic repair. For this reason, previous RP is regarded as a contraindication to LIHR. Most surgeons advocate an open, anterior approach, avoiding the prostatectomy-related preperitoneal fibrotic reaction that can make dissection in this plane challenging. LIHR repair offers many advantages over the open technique, including shorter hospital stay, faster post-operative recovery and return to normal daily activities, reduced pain and improved cosmesis [5]. To date fewer than 100 LIHR + RP operations utilising the TEP method have been published in the literature [2, 6].

The primary aim of this study was to compare the outcome of patients undergoing TEP LIHR post RP to a matched control group undergoing LIHR without prior groin surgery. The secondary aim was to compare the outcome of patients relative to the RP approach.

## Materials and methods

### Study design and setting

This retrospective case–control study analysed the outcome of LIHR using the TEP approach in the context of previous RP. LIHR post RP was compared to primary LIHR operations between 1995 and 2020, using information obtained from the prospective database of a single surgeon at a private surgical unit in Auckland, Aotearoa New Zealand. Institutional approval was obtained for the study.

**Fig. 1:**
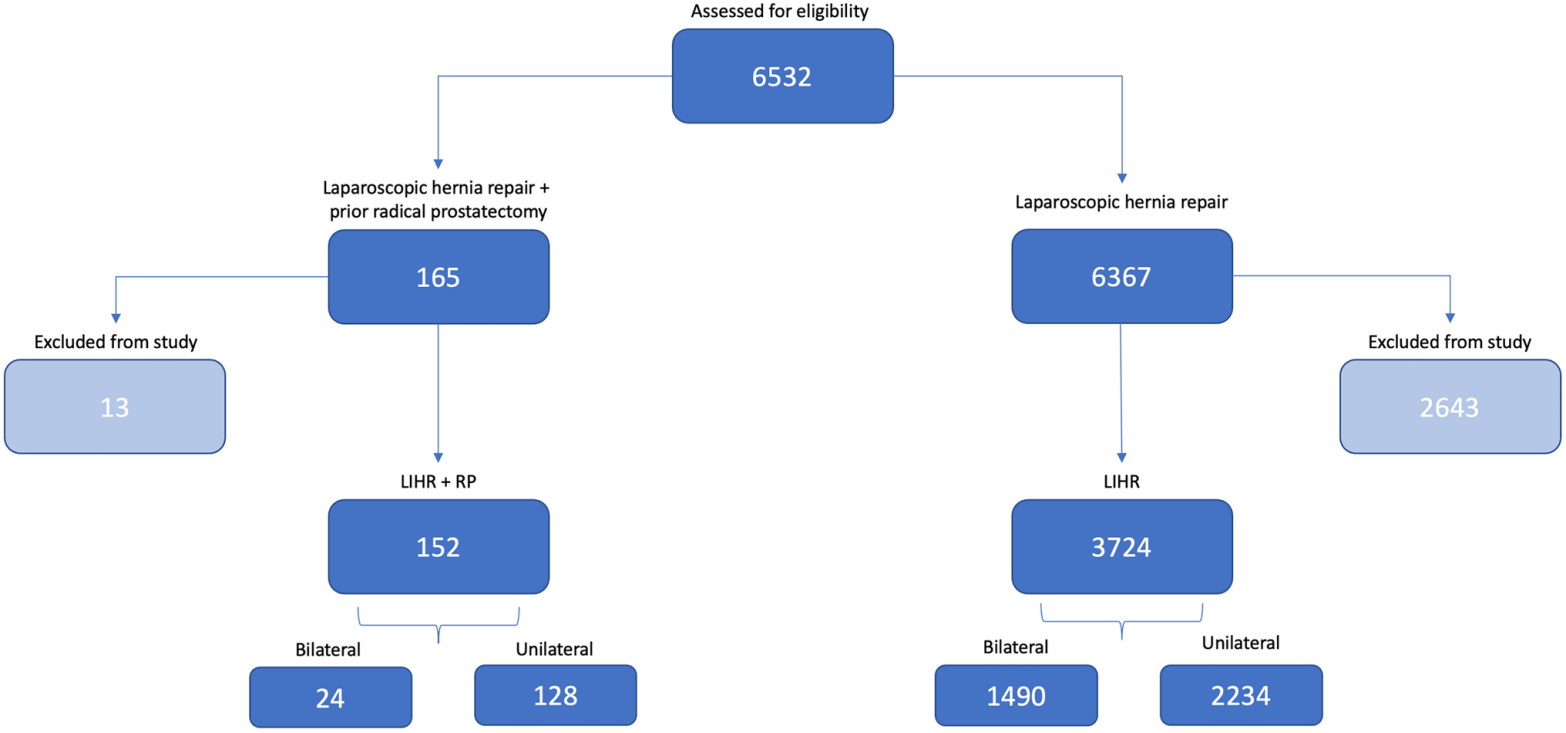
Consort diagram of patients undergoing TEP LIHR

A total of 6532 laparoscopic hernia repair operations were identified and assessed for eligibility (Fig. 1). Operations were divided into two groups. The first group (*n* = 165) included LIHR post RP (LIHR + RP). The control group (*n* = 6367) included primary LIHR operations only. Patients undergoing unilateral and bilateral LIHR were included. Bilateral repair was counted as two hernia repair operations and compared like for like in the analysis.

### Inclusions and exclusions

Exclusion criteria: females, non-inguinal hernia repair operations (femoral hernia), chronic groin pain without herniation (groin strain/sportsman’s hernia), trans-abdominal (TAPP) approach, patients undergoing a concurrent additional operation which might confer an increase in morbidity and, for the LIIHR group, age ranges outside of age < 50 or > 87 years. Exclusion of ages outside of this range aligned the LIHR group with the age range of the LIHR + RP group, reducing any potential confounding factors. Operations were excluded in the sequence detailed above to ensure an excluded operation was only counted once.

In the LIHR + RP group, 13 operations were excluded (9 unilateral, 4 bilateral). Exclusions were for the following: femoral hernia (1), groin strain/sportsman’s hernia (2), additional surgical procedure (8) and a TAPP approach (2). The remaining 152 LIHR + RP operations were included in the analysis, 128 unilateral and 24 bilateral (140 patients with prior RP). The types of initial RP techniques utilised were open (93 of 140), robotic (18 of 140) or laparoscopic (17 of 140) with 12 patients having unspecified type of prior RP repair (Table 2).

In the LIHR group, 2643 operations were excluded (1907 unilateral, 736 bilateral). Exclusions were for the following: female patient (340), femoral hernia (66), groin strain/sportsman’s hernia (225), a TAPP approach (39), additional surgical procedure (244) and age < 50 or > 87 years (1729). This left 3724 LIHR operations included in the analysis 2234 unilateral and 1490 bilateral (2979 patients).

### Surgical technique

Access to the extraperitoneal plane was gained through a short vertical incision in the ipsilateral anterior rectus sheath just below the level of the umbilicus, with introduction of a 0° 10 mm laparoscope. Two 5 mm operating ports were placed in the lower abdominal midline below the umbilicus, via the linea alba. Balloon dissection was not used to reduce the chance of peritoneal tearing against post-RP scarring. The extraperitoneal space was developed working from lateral, where tissues have not been previously dissected, to medial, where RP scarring is anticipated. A combination of sharp and blunt dissection was employed, taking care to dissect against the posterior wall of the inguinal canal in areas of scarring, in order to avoid inadvertent injury of any adherent bladder or bowel. Considerable care was exerted when dissecting close to the iliac vessels. A 15 × 10 cm polypropylene mesh was fixed to the periosteum of the superior pubic ramus and the linea alba using penetrative titanium tacs. Fixation laterally was not employed in order to allow any subsequent mesh contraction to occur without impediment. The aim was for day stay surgery. Patients were encouraged to return to full activity, without any restrictions regarding lifting/straining. Follow-up consisted of clinical review in 10–14 days and telehealth consultation at 3 months. The surgeon’s mobile phone number was provided for ease of contact if needed.

### Variables analysed

Quantitative and qualitative pre-operative, operative and post-operative variables were assessed immediately prior to surgery, at operation, at clinical follow-up (10–14 days post-operatively) and by telehealth 3 months post-operatively. Pre-operative variables included age, sex and body mass index (BMI). Clinical variables assessed by the surgeon included hernia location (right or left), size (small, medium or large), whether the hernia was direct or indirect, and if the patient had previously undergone hernia repair. Operative variables included the type of hernia, presence of lipoma of the spermatic cord, duration of operation (incision to wound closure), perceived difficulty of operation by surgeon (visual analogue score of 1–10) and details of any concurrently performed surgical procedure. Post-operative variables examined included hospital stay (classified as discharged on day of surgery, the day following surgery or > 48 h) and any post-operative complications or readmission. Patient perceived variables included the number of days until return to normal function, return to work, resumption of driving and the number of days simple analgesia was required (paracetamol or non-steroidal anti-inflammatory drugs). At three months patients had phone call follow-up and were asked if they had any residual awareness of the repair and if they had any restriction in movement, or function, as a result of their operation. Awareness and restriction were classified by the patient as nil, mild, moderate or severe. Any presence of hernia recurrence was also recorded. Clinical review was offered to all patients.

### Statistical analysis

Descriptive statistics were used to characterise the groups, with mean and standard deviation (SD), or median and range. Groups were compared using Fisher’s Exact and Chi-squared test for categorical data, the Mann–Whitney test for non-parametric numerical data, unpaired *T*-tests for parametric numerical data and the Kruskal–Wallis and Dunn’s test for multiple comparisons (GraphPad Prism 8.40, USA). A *p* value < 0.05 was considered statistically significant.

## Results

### Pre-operative variables: (Tables 1 and 2, Fig. 2)

**Table 1:** Pre-operative variables

Pre-operative demographics				
	LIHR		LIHR + RP	
	Unilateral	Bilateral	Unilateral	Bilateral
Age (median)	62 (IQR = 14)	62 (IQR = 14)	68 (IQR = 9.8)	71 (IQR = 11.8)
Weight, kg (median)	80	79	78	78
BMI (median)	26	26	26	26
Previous hernia repair	235 (10.5%)	34 (2.3%)	24 (17.1%)	1 (4.2%)
Previous hernia recurrence	208 (9.3%)	137 (9.2%)	6 (4.3%)	1 (4.2%)
Total IHR	2234 (100%)	1490 (100%)	140 (100%)	24 (100%)

States the relevant numbers (either median and interquartile range, or whole numbers and percentage of total) for the pre-operative demographics of age, weight, BMI, previous hernia repair and previous hernia recurrence

**Table 2:** LIHR + RP initial prostatectomy technique

Radical prostatectomy: initial operation technique			
	Unilateral	Bilateral	Unliteral + Bilateral
Number of IHR	128	24	152
Open	86	7	93 (66%)
Robotic	16	2	18 (13%)
Laparoscopic	14	3	17 (12%)
Unknown/Unspecified	12	0	12 (9%)
Number of prior RP	128	12	140 (100%)

States the original technique—open, laparoscopic, robotic or unknown—of initial RP surgeries. This is shown as a whole number and percentage of total LIHR + RP operations for both unilateral and bilateral groups. Note that the 24 bilateral LIHR + RP operations are counted as 12 previous RP operations

**Fig. 2:**
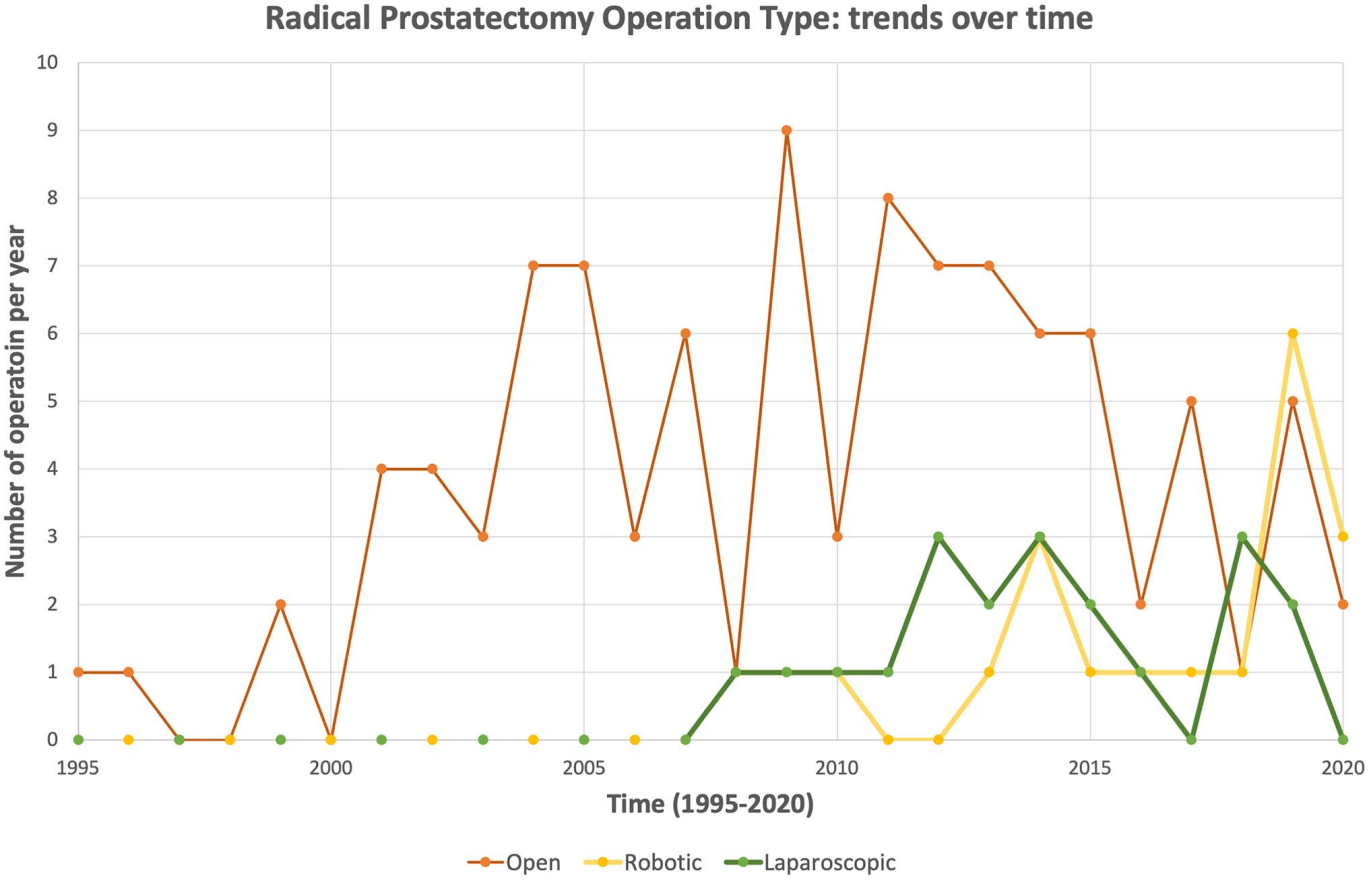
Radical prostatectomy operation type: trends over time. A line graph of which prostatectomy technique was utilised, open, robotic or laparoscopic, in the operations included in the TEP LIHR + RP group. The year recorded is of hernia repair, not the year the radical prostatectomy, as date of original operation was not always available, resulting in trend lines being shifted out of phase

There was a difference in age (*p* < 0.0001) between LIHR and LIHR + RP. The median age was 62 years for both unilateral (IQR = 14y) and bilateral (IQR = 11y) LIHR, compared to 68 for unilateral (IQR = 9.8y) and 71 for bilateral (IQR = 11.8y) LIHR + RP. There was no difference in BMI (*p* = 0.2563 unilateral, *p* = 0.7379 bilateral) or weight (*p* = 0.2295 unilateral, *p* = 0.4079 bilateral) between LIHR and LIHR + RP groups. There were differences in the rates of previous hernia repair with 17.1% of unilateral LIHR + RP operations identified as having a previous hernia repair on the contralateral side, compared to 10.5% for unilateral LIHR (*p* = 0.0079). There was no difference in rates of previous hernia recurrence (*p* = 0.0658 unilateral, *p* > 0.9999 bilateral).

Previous RP technique was initially dominated by the open method with 66% (93 of 140) undergoing open RP, 13% (18 of 140) robotic and 12% (17 of 140) laparoscopic (Table 2). Previous RP technique was not specified in 9% (12 of 140). As the study period progressed, robotic RP became the dominant contributor (Fig. 2).

### Operative variables: (Tables 3 and 4, Fig. 3)

**Table 3:** Operative variables

Operative variables				
	LIHR		LIHR + RP	
	Unilateral	Bilateral	Unilateral	Bilateral
Conversion to open	0 (0%)	0 (0%)	3 (2.1%)	0 (0%)
Operative time, minutes	21 (IQR 9–120)	30 (IQR 16–105)	40 (IQR 20–114)	71 (IQR 31–111)
Right hernia	1263 (56.5%)	745 (50%)	74 (52%)	12 (50%)
Left hernia	971 (43.5%)	745 (50%)	66 (48%)	12 (50%)
Lipoma	238 (10.7%)	142 (9.5%)	11 (7.9)	4 (16.7%)
Total IHR	2234 (100%)	1490 (100%)	140 (100%)	24 (100%)

States the relevant numbers (either median and interquartile range, or whole number and percentage of total) for the operative variables of conversion to open, operative time (in minutes), side of herniation and presence of lipoma

**Table 4:** LIHR + RP operative variables

Radical prostatectomy			
	Open	Robotic	Laparoscopic
Operative time, minutes (unilateral only)	40 (IQR 20–114)	39.5 (IQR 22–100)	59 (IQR 20–74)
Conversion to open (unilateral + bilateral)	2 (2.2%)	0 (0%)	0 (0%)
LIHR + RP (unilateral + bilateral)	93 (100%)	18 (100%)	17 (100%)

States the operative variables of operation time (in minutes) and conversion to open, in the LIHR + RP group, against initial RP type (open, robotic or laparoscopic)

**Fig. 3:**
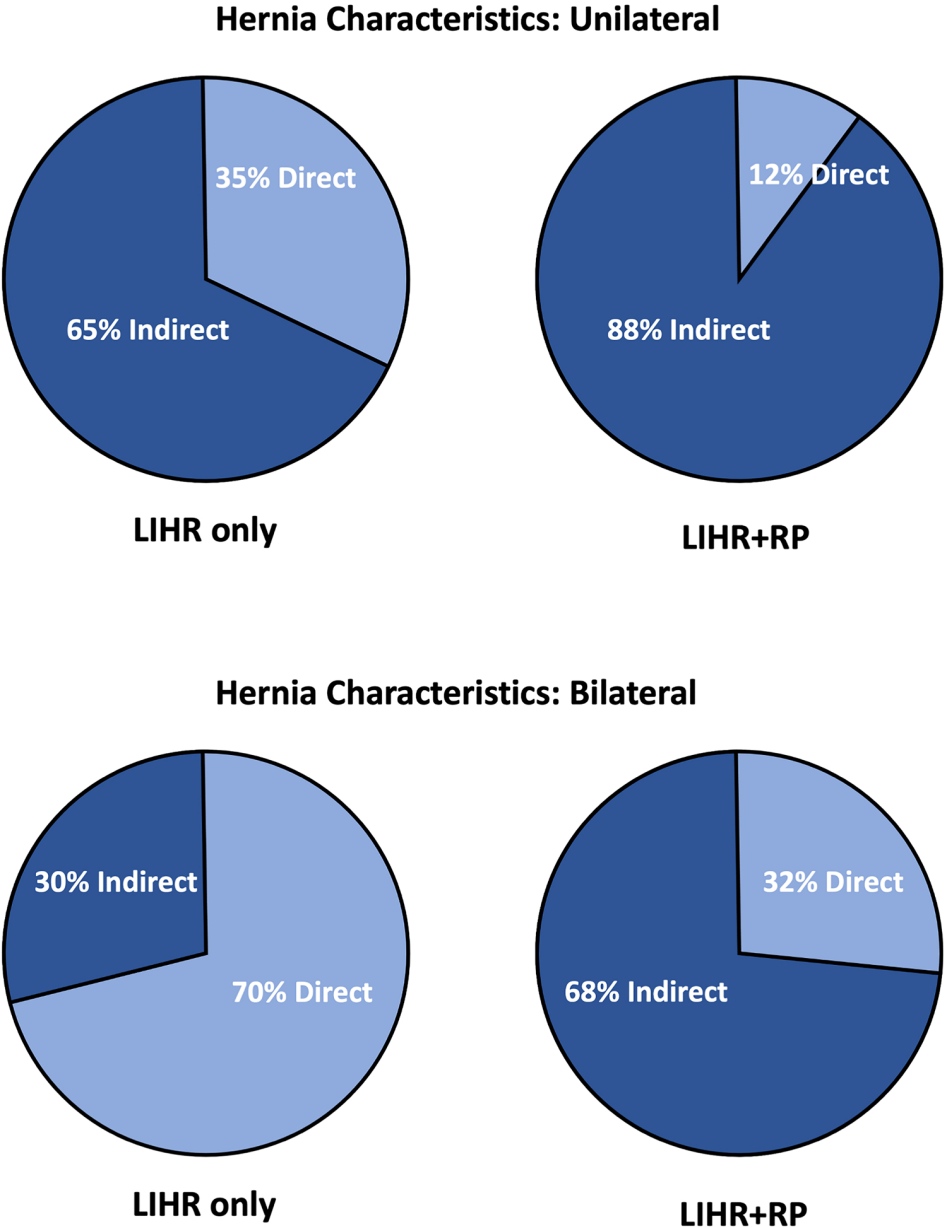
Hernia characteristics. It is a pie graph demonstrating the hernia characteristic in the groups, simple laparoscopic inguinal hernia repair operations compared to laparoscopic inguinal hernia repair operations in the context of prior radical prostatectomy. This is further stratified by bilateral and unilateral herniations to allow for accurate comparative analysis. Inguinal hernia characteristics were recorded by the surgeon as indirect or direct

Three of the first 13 cases in the LIHR + RP series required conversion to open. These have been excluded from results analysis when looking at outcomes of (completed) LIHR + RP. The median operative time was 21 min (range 9–120) for unilateral LIHR and 40 min (range 20–114) for unilateral LIHR + RP, a 19-min difference (*p* < 0.0001). The median operative time was 30 min (range 16–105) for bilateral LIHR and 71 min (range 31–111) for bilateral LIHR + RP, a 41-min difference (*p* < 0.0001). Operative time between open, robotic and laparoscopic unilateral LIHR + RP displayed a difference (*p* = 0.0336), with a median of 40 min (range 20–114) for open RP, 39.5 min (range 22–100) for robotic RP and 59 min (range 20–74) for laparoscopic RP. Comparison analysis by RP technique demonstrated a difference in operative time between laparoscopic vs. open (*p* = 0.0355) but no difference between laparoscopic vs. robotic (*p* = 0.0937), or open vs. robotic (*p* > 0.9999).

There was no difference in hernia size (*p* = 0.1563 unilateral, *p* = 0.2308 bilateral), side of herniation in unilateral cases (*p* = 0.7288) or element of lipoma (*p* = 0.5544 unilateral, *p* = 0.2808 bilateral) between LIHR and LIHR + RP. Indirect hernias were more common in the LIHR + RP group, 88% of unilateral and 68% of bilateral, compared to the LIHR group, 65% and 30%, respectively (Fig. 3).

### Post-operative variables: (Tables 5, 6, 7)

**Table 5:** Post-operative variables, recovery parameters

Post-operative variables: immediate recovery parameters				
	LIHR		LIHR + RP	
	Unilateral	Bilateral	Unilateral	Bilateral
Discharge the day of surgery	2203 (98.6%)	1458 (97.9%)	132 (94.5%)	22 (91.7%)
Complications	65 (2.9%)	24 (1.6%)	3 (2.1%)	2 (8.3%)
Return to normal function (days)	3.5 (IQR 0–27)	3.8 (IQR 0–18)	3.5 (IQR 0–10)	5.1 (IQR 0–14)
Return to driving (days)	2.2 (IQR 0–11)	2.3 (IQR 0–10)	2.6 (IQR 0–10)	2 (IQR 1–3)
Analgesia use (days)	2.2 (IQR 0–21)	2.6 (IQR 0–14)	2.7 (IQR 0–16)	3.2 (IQR 0–10)
Total IHR	2234 (100%)	1490 (100%)	140 (100%)	24 (100%)

States the relevant numbers, either median and interquartile range, or whole number and percentage of total for the post-operative variables of time to discharge, complications, return to normal function, return to driving and analgesia use

**Table 6:** Post-operative variables, three-month follow-up

Post-operative variables: three-month follow-up				
	LIHR		LIHR + RP	
	Unilateral	Bilateral	Unilateral	Bilateral
Initial group	2234	1490	140	24
Retained at 3 months	2138 (95.7%)	1341 (90%)	124 (88.6%)	18 (75%)
Lost to follow-up	96 (4.3%)	149 (10%)	16 (11.4%)	6 (25%)
No awareness	1911 (89.4%)	1218 (90.8%)	114 (92%)	16 (88.9%)
No restriction	2117 (99%)	1341 (100%)	124 (100%)	18 (100%)
Total IHR in follow-up	2138 (100%)	1341 (100%)	124 (100%)	18 (100%)

States the whole numbers and percentage total of the group, for the three-month post-operative variables of lost to follow-up, awareness and restriction. Awareness and restriction are analysed as percentages from the total of those retained at three-month follow-up

**Table 7:** Unilateral post-operative variables by radical prostatectomy technique, three-month follow-up

Radical prostatectomy: three-month follow-up (unilateral + bilateral)			
	Open	Robotic	Laparoscopic
Initial group	93	18	17
Retained at 3 months	82 (88.2%)	16 (88.9%)	15 (88.2%)
Lost to follow-up	11 (11.8%)	2 (11.1%)	2 (11.8%)
No awareness	76 (92.7%)	14 (87.5)	14 (93.3%
No restriction	82 (100%)	16 (100%)	15 (100%)
Total LIHR + RP in follow-up	82 (100%)	16 (100%)	15 (100%)

States the whole numbers and percentage total, for three-month post-operative variables in the LIHR + RP group, against initial RP type—open, robotic or laparoscopic. Awareness and restriction are analysed as percentages from the total of those retained at three-month follow-up

Time to discharge was recorded as day of surgery, the day following surgery or > 48 h post surgery, with no difference between LIHR and LIHR + RP (unilateral *p* > 0.9999, bilateral *p* = 0.0517). The majority were discharged on the day of surgery, 98.6% of unilateral LIHR, 97.9% of bilateral LIHR, 94.5% of unilateral LIHR + RP and 91.7% of bilateral LIHR + RP. Only 10 patients (0.26%) were discharged at > 48 h, none LIHR + RP. Complication rates were not different between LIHR and LIHR + RP (unilateral *p* = 0.2840, bilateral *p* = 0.0621). Complications (e.g. hematoma or superficial wound infection) occurred in 2.9% of unilateral LIHR, 1.6% of bilateral LIHR, 2.1% of unilateral LIHR + RP and 8.3% of bilateral LIHR + RP. No complications resulted in loss of life or serious morbidity. Return to normal function was not different between LIHR and LIHR + RP (unilateral *p* = 0.9034, bilateral *p* = 0.4025), with a mean of 3.5 days (range 0–27) for unilateral LIHR, 3.8 days (0–18) for bilateral LIHR, 3.5 days (0–10) for unilateral LIHR + RP and 5.1 days (0–14) for bilateral LIHR + RP. Return to driving was not different between LIHR and LIHR + RP (unilateral *p* = 0.0513, bilateral *p* = 0.9320), with a mean of 2.2 days (range 0–11) for unilateral LIHR, 2.3 days (0–10) for bilateral LIHR, 2.6 days (0–10) for unilateral LIHR + RP and 2 days (1–3) for bilateral LIHR + RP were reported. Analgesic use was not different between LIHR and LIHR + RP (unilateral *p* = 0.1328, bilateral *p* = 0.7738), with a mean of 2.2 days (range 0–21) for unilateral LIHR, 2.6 days (0–14) for bilateral LIHR, 2.7 days (0–16) for unilateral LIHR + RP and 3.2 days (0–10) for bilateral LIHR + RP.

Awareness and restriction were assessed categorically three months post-op, as no awareness versus awareness, and no restriction versus restriction, using the Chi-squared test. Losses to follow-up were 4.3% of unilateral LIHR, 10% of bilateral LIHR, 11.4% of unilateral LIHR + RP and 25% of bilateral LIHR + RP. There was no difference in awareness between LIHR and LIHR + RP (unilateral *p* = 0.3689, bilateral *p* = 0.0538), with 89.4% of unilateral LIHR, 90.8% of bilateral LIHR, 92.0% of unilateral LIHR + RP and 88.9% of bilateral LIHR + RP reporting no awareness. There was no difference in awareness by RP technique, with awareness reported in 7.3% of open, 12.5% of robotic and 6.7% of LIHR + RP (*p* = 0.7671). There was a difference in restriction between unilateral LIHR and LIHR + RP, with restriction reported in 0.06% of LIHR vs. 0% of LIHR + RP (*p* = 0.0006), but no difference between bilateral LIHR and LIHR + RP with no restriction reported in either group (*p* = 0.0563). There was no difference in restriction by RP technique, with no restriction reported after any of the LIHR + RP operations. Patients reported any awareness or restriction as mild, moderate or severe, with no patients reporting these as moderate or severe.

There was no difference in hernia recurrence between LIHR and LIHR + RP (unilateral *p* = 0.0658, bilateral *p* =  > 0.9999). Recurrence was reported in 0.27% of unilateral LIHR (6 of 2234), 0.2% of bilateral LIHR (3 of 1490), 1.6% of unilateral LIHR + RP (2 of 128) and 0% of bilateral LIHR + RP cases (0 of 24). Of the two LIHR + RP recurrences, the first was considered a recurrence clinically, but on surgical exploration consisted of a small lipoma only. If this was excluded, unilateral LIHR + RP recurrence decreases to 0.78% (one in 128).

## Discussion

Previous RP, whether open, laparoscopic or robotic, should not be considered an absolute contraindication to TEP LIHR. In this series, no difference in morbidity, mortality or recovery was demonstrated when comparing LIHR and LIHR + RP, for either unilateral or bilateral repairs.

These findings are supported by the limited literature available on TEP inguinal hernia repairs. One systematic review published in 2019 [6] and two prospectively collected case–control studies that included TEP hernia repair in patients with previous RP were identified [7, 8]. These included a total of 277 LIHR operations (229 patients) in the context of previous RP and included both unilateral and bilateral repairs (181 unilateral and 48 bilateral) [6]. The majority of operations in these papers were performed using the TAPP approach, only 62 had a TEP repair [6]. The 2019 systematic review reported no difference in post-operative complications, conversions to open or hernia recurrence between TEP and TAPP operative methods [6]. The first case–control study looked at LIHR operations performed after RP or previous lower abdominal surgery, matched to patients without previous surgery and included 10 TEP LIHR + RP operations from a total of 256 LIHR operations [7]. It concluded that, while TEP LIHR repairs post RP have a longer operative time when compared to LIHR without prior RP, they can be performed efficiently and safely by experienced laparoscopic surgeons [7]. The second case–control study reviewed outcomes for 52 TEP LIHR + RP operations, matched to a control group who had not undergone RP [8]. It concluded that TEP LIHR post RP is safe, with equivalent outcomes to LIHR alone, and that the slightly longer operative times compared to an open hernia repair may be justified by early discharge and reduced post-operative pain with LIHR [8].

While publications on LIHR + RP are currently dominated by the TAPP method, there is no evidence supporting the superiority of the TAPP or TEP approach to hernia repair [2, 6, 9–13]. Multiple, large, published analyses have found TEP and TAPP comparable for all important post-operative outcomes including length of hospital stay, infection, hernia recurrence and chronic pain [2, 10–14]. The choice of hernia repair technique, TEP or TAPP is a matter of surgeon experience and preference [2, 10, 11]. The surgeon of this series started performing LIHR using the TAPP technique, switching to his preferred TEP approach in 1995. The results of this paper in conjunction with the published literature demonstrate that both TEP and TAPP approaches are safe in the context of previous RP [2, 6, 9–11]. From this literature review, it appears that our series, which includes 152 cases of TEP LIHR + RP, is the largest study of TEP LIHR + RP currently published with only 62 other TEP LIHR + RP cases found in the literature. Publications on TAPP LIHR + RP only support our conclusion that TEP LIHR post RP can be performed safely.

There is a significant learning curve to LIHR, whether TAPP or TEP is used. LIHR following RP confers an extra layer of potential difficulty due to scarring of the extraperitoneal plane over the posterior wall of the inguinal canal. This is reflected in the surgeon of this series needing to convert three of his first 13 LIHR + RP cases to open, despite having done nearly 400 LIHR before embarking on his first LIHR + RP case. He recommends that any decision to undertake LIHR post RP should only be made once a high level of competency with LIHR has been attained. There is no set number of cases to achieve competency. Surgical ego should not prevent sensible decision-making regarding conversion to, or selection of, open repair. The outcome of longer operative time for LIHR + RP in this series reflects the more challenging surgical environment post-RP secondary to RP scarring, even in experienced hands. While there is increased difficulty in performing LIHR + RP, these results support that this is a hernia repair that can be performed by the experienced laparoscopic surgeon [15–17].

The dominance of indirect over direct inguinal hernias in the LIHR + RP group is explained by post-RP scarring, which is usually confined to the posterior wall of the inguinal canal, medial to the deep inguinal ring (Fig. 3). This scarring is an impediment to direct hernia formation, but indirect herniation into the inguinal canal is relatively unimpeded.

Although the surgical literature considers RP a major risk factor for inguinal hernia formation, in this series 18.75% of unilateral LIHR + RP patients had a previous IHR on the contralateral side, compared to 10.47% of LIHR alone [2], despite the two groups being matched for age and other controls. These results suggest that an underlying susceptibility to hernia development may also be a contributing factor to hernia formation post RP, contrasting with the current surgical perception.

The results of this series allowed for some comparison of the impact that the initial RP surgical technique—open, laparoscopic, or robotic—has on subsequent LIHR outcomes. Although the numbers in this study are limited, they suggest that initial RP technique does not impact on post-operative variables of awareness, restriction or recurrence in LIHR. The amount and density of scarring post RP varied from case to case, with no apparent influence by original RP technique. As in many parts of the world, robotic RP is becoming the dominant technique in Aotearoa New Zealand, but robotic RP did not appear to confer any increased ease of subsequent LIHR compared to open or laparoscopic, though the numbers in this series are very small for meaningful analysis. We acknowledge that these are findings that would need larger numbers and further research to form any definitive conclusions, but these results do seem promising that all patients who have undergone prior RP, regardless of RP type, should be considered for LIHR. To our knowledge, this is the first publication to look at the impact of initial RP type on subsequent hernia repair.

While this study has several limitations, it also demonstrates many strengths. Its largest limitation is that it is based off observational retrospective data. It is also limited in that there was only three-month follow-up and it is plausible that some hernia recurrence, an important post-operative variable, was missed consequently. However, the variable being examined, prior RP, would make an RCT-based study impossible on ethical and practical grounds and all similar studies of this topic by nature will be observational retrospective studies. The large number of cases, 3876 overall, 152 LIHR + RP, gives this study strength and reduces the risk of bias despite its observational structure. Using data from a single surgeon, over a period of 25 years, removes intra-operative variability due to the surgical techniques and variances between surgeons.

In summary, previous RP should not be considered an absolute contraindication for LIHR. Although previous RP adds complexity, in experienced hands TEP LIHR can be done safely, with outcomes being equivalent to patients who have not previously undergone RP.
